# Forensic profiling of smokeless powders (SLPs) by gas chromatography–mass spectrometry (GC-MS): a systematic investigation into injector conditions and their effect on the characterisation of samples

**DOI:** 10.1007/s00216-024-05189-w

**Published:** 2024-02-09

**Authors:** Blake Kesic, Niamh McCann, Samantha L. Bowerbank, Troy Standley, Jana Liechti, John R. Dean, Matteo D. Gallidabino

**Affiliations:** 1 Department of Applied Sciences, Northumbria University, Newcastle Upon Tyne, NE1 8ST UK; 2 King’s Forensics, Department of Analytical, Environmental & Forensic Sciences, King’s College London, London, SE1 9NH UK

**Keywords:** Forensics/toxicology, Explosives, GC, Chemometrics/statistics, Chemical profiling, Smokeless powder

## Abstract

Smokeless powders (SLPs) are composed of a combination of thermolabile and non-thermolabile compounds. When analysed by GC-MS, injection conditions may therefore play a fundamental role on the characterisation of forensic samples. However, no systematic investigations have ever been carried out. This casts doubt on the optimal conditions that should be adopted in advanced profiling applications (e.g. class attribution and source association), especially when a traditional split/splitless (S/SL) injector is used. Herein, a study is reported that specifically focused on the evaluation of the liner type (*L*_type_) and inlet temperature (*T*_inj_). Results showed that both could affect the exhaustiveness and repeatability of the observed chemical profiles, with *L*_type_ being particularly sensitive despite typically not being clarified in published works. Perhaps as expected, degradation effects were observed for the most thermolabile compounds (e.g. nitroglycerin) at conditions maximising the heat transfer rates (*L*_type_ = packed and *T*_inj_ ≥ 200 °C). However, these did not seem to be as influential as, perhaps, suggested in previous studies. Indeed, the harshest injection conditions in terms of heat transfer rate (*L*_type_ = packed and *T*_inj_ = 260 °C) were found to lead to better performances (including better overall %RSDs and LODs) compared to the mildest ones. This suggested that implementing conditions minimising heat-induced breakdowns during injection was not necessarily a good strategy for comparison purposes. The reported findings represent a concrete step forward in the field, providing a robust body of data for the development of the next generation of SLP profiling methods.

**Graphical abstract:**

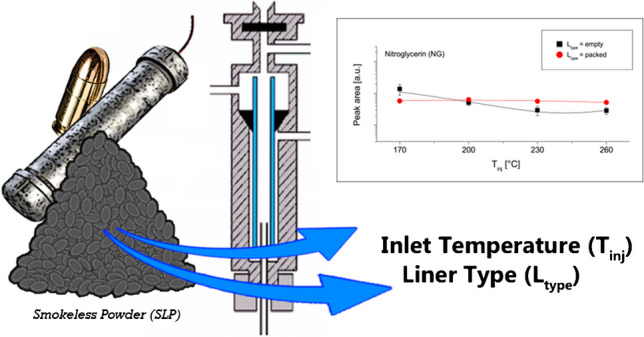

**Supplementary Information:**

The online version contains supplementary material available at 10.1007/s00216-024-05189-w.

## Introduction

Smokeless powders (SLPs) are energetic materials that are typically used as propellants in firearms or artillery ammunition [1]. Due to their relative ease of accessibility, however, they are also very often employed illegally as main charges in improvised explosive devices (IEDs), such as pipe bombs [2]. As a consequence, SLPs are commonly encountered in the investigation of many firearm- and explosive-related crimes, making their examination, analysis and profiling, very important from a forensic perspective. These procedures, indeed, may provide information to help investigators identify the brand of a SLP used in a seized IED (i.e. class attribution) [3, 4] and/or establish a link between two different SLPs seized at different locations (i.e. source association) [5, 6].

Smokeless powders are chemical mixtures that include a range of organic compounds, such as explosives (e.g. nitrocellulose and nitroglycerin), stabilisers (e.g. diphenylamine and ethyl centralite) and plasticisers (e.g. dibutyl phthalate and diethyl phthalate) [1, 7]. These compounds are typically conditioned in grains that are manufactured in a wide range of colours, shapes and sizes, in order to meet specific ballistic properties [8, 9]. Therefore, the standard procedure for SLP examination typically starts with a study of the physical aspect of the grains (and, eventually, their absorption/luminescence properties) by visual and optical techniques [9]. Chemical analysis is then carried out on a test portion. For this purpose, many techniques have been proposed, including vibrational spectroscopic methods, such as Fourier-transform infrared spectroscopy (FTIR) and Raman spectroscopy [10–12], as well as separation methods, such as capillary electrophoresis (CE), liquid chromatography (LC) and gas chromatography (GC) [5, 13–15]. Amongst them, GC presents the unique advantage that it is widespread in forensic laboratories and offers a high degree of selectivity for the main SLP compounds, especially in hyphenation with mass spectrometry (MS) detection. Hence, it rapidly became a method of choice, with many reported uses in published works, guidelines and standard methods. ASTM International, for example, explicitly suggests the use of GC for SLP analysis in the guideline E2998 [16] and also describes a series of suggested requirements for a GC-MS method in the guideline E2999 [17].

Despite being a largely accepted technique for SLP analysis, GC involves the use of heat to achieve volatilisation of the analytes, which is an essential requirement for their column introduction, separation and, eventually, transfer to MS. This means that its implementation is not without challenges. Indeed, many SLP compounds, such as nitroglycerin and nitro-diphenylamine, are thermolabile and can undergo breakdowns during the analysis, with losses in their limits of detection and other degradation effects [5, 18]. The injection conditions are particularly influential [19], and a number of *ad hoc* strategies have been proposed over the years to minimise the heat transfer rate during the column introduction step. These can be classified into two general approaches: (1) the application of a temperature gradient using a programmable temperature vaporising (PTV) injector or (2) the application of a mild isothermal temperature using a traditional split/splitless (S/SL) injector. While the first approach is surely the most promising, PTV injectors are expensive and delicate and, as a consequence, still not very common in forensic laboratories. Therefore, since the first pioneering works of Mach *et al.* [20, 21] in 1978, the second approach has become increasingly popular amongst practitioners, especially in combination with a low boiling point injection solvent and/or an empty liner instead of a packed one (to further reduce heat transfer rates). The ASTM guideline E2999, for instance, explicitly suggests the use of a S/SL injector with an inlet temperature between 190 and 220 °C, as well as acetone or dichloromethane as injection solvents [17]. Similar conditions were adopted by many research works, often by further reducing the inlet temperature down to 170 °C [22].

While the previous conditions proved beyond any reasonable doubt their suitability for the forensic analysis of SLPs, they can also bring, at least on a theoretical level, a number of non-negligible side effects. Both the use of a low liner temperature and an empty liner, for example, typically lead to slow inlet-to-column mass transfer rates which, in turn, could affect the precision of the observed signals, as well as the volatilisation efficiency of the less volatile compounds [19]. The most advanced forensic profiling applications (e.g. class attribution and source association, as described above) would surely benefit from enhanced measurement precision and reduced analyte discrimination at the column introduction step. Perhaps surprisingly, however, very little information is available in published literature to date regarding the effects of the aforementioned parameters on the characterisation of SLP samples and, especially, on the further comparison of extracted chemical profiles. The liner type, in particular, is rarely reported in the experimental sections of most published works, which indicates its general underestimation. This situation makes selection of the most appropriate injection conditions for specific applications very challenging, in addition to casting doubt on the actual suitability of current mainstream strategies for SLP analysis in wider profiling applications.

The purpose of this work was to address this gap. Two parameters were specifically evaluated: the liner type (*L*_type_) and inlet temperature (*T*_inj_). Firstly, their effects on the responses of 12 compounds commonly found in SLPs were investigated. The two set of injection conditions (SICs) leading to the milder and larger heat transfer rates were then selected and compared in terms of analytical performances. Finally, the two SICs were applied to the analysis of ten different SLPs, in order to evaluate their respective suitability for sample comparison. Chemometric approaches were applied for the latter assessment. This is the first time that a systematic evaluation of the main parameters affecting the injection of SLP samples in GC has been carried out. It is also the first time that method evaluation has been extended, at least in SLP analysis, to practical profiling applications through chemometric methods.

## Experimental

### Chemicals and materials

Methanol (MeOH) and dichloromethane (DCM) were purchased from Thermo Fisher Scientific (Loughborough, UK) and were both of HPLC grade (> 99.9%). A total of 12 compounds typically used in SLP formulations were selected as target analytes, and standard reference materials were obtained from different chemical companies (see details in the Electronic Supplementary Material, Table S1). The analytes were (in elution order): nitroglycerin (NG), 2,6-dinitrotoluene (26DNT), 2,4-dinitrotoluene (24DNT), diethyl phthalate (DEP), diphenylamine (DPA), methyl centralite (MC), ethyl centralite (EC), dibutyl phthalate (DBP), 2-nitrodiphenylamine (2NDPA), akardite II (AK2), 4-nitrodiphenylamine (4NDPA), and 2,4-dinitrodiphenylamine (DNDPA). Phenanthrene-d10 (PHE) was also purchased and used as internal standard.

For each substance, an individual stock solution was prepared at a concentration of 1000 mg L^−1^ in MeOH, with the exception of DNDPA for which DCM was used instead to guarantee complete dissolution. Stock solutions were stored in the dark (freezer) at −20 °C. Working solutions were freshly prepared daily by dilutions of stock solutions with DCM. Quality control (QC) and internal standard (IS) spiking solutions were also freshly prepared daily in the same solvent as the other samples in the sequence. The QC solution contained all the target analytes (IS included) at a concentration of 10 mg L^−1^, with the exception of NG and DNDPA, which were included at a concentration of 100 mg L^−1^. The IS spiking solution only contained PHE at a concentration of 100 mg L^−1^.

### GC-MS equipment

All experiments were performed using a Thermo Trace 1330 C gas chromatograph (Thermo Scientific, Loughborough, UK), coupled to a Thermo ISD 7000 mass selective detector and fitted with a Thermo TG-5MS column, 30 m × 0.25 mm × 0.25 μm. The instrument was equipped with a Thermo AS 1310 autosampler.

The effect of the liner type and inlet temperature were evaluated and, therefore, varied between the experiments (as described in the corresponding section below). All other instrumental parameters were consistent and based on common practices in the field [5, 22–24]. Injection was carried out in split mode, with a 25:1 split ratio, and the injection volume was set to 1 μL. The carrier gas was helium, and the column flow was maintained at 1.2 mL min^−1^. The oven ramp was programmed as follows: 40 °C for 1 min, increased to 280 °C at 20 °C min^−1^ and held at this temperature for 7 min (total chromatographic time: 20 min). The transfer line between the column and the MS was held at 260 °C. Ionisation was carried out through electron impact (EI). Masses were scanned from *m/z* 40 to 300. Solvent delay was set to 4.5 min, and MS source temperature was set to 230 °C. A post-run conditioning step was included after each analysis. For this, the column flow was increased to 3.0 mL min^−1^ and the column temperature to 310 °C for 5 min. No carryovers were observed with this method at any of the conditions tested, including at the lowest inlet temperatures. QC samples were used within each run to check the stability of the analyte responses over time. These were injected every five samples.

### Injection parameters

Two liner types (with and without quartz wool, also referred to as packed and empty) and four inlet temperatures (170, 200, 230 and 260 ºC) were tested. Both the liner types were purchased from Thames Restek (High Wycombe, UK) and were straight split liners, 4 × 6.3 × 78.5 mm (ID × OD × L). The liners with quartz wool were prepacked.

All optimisation tests were carried out *via* injection of eight different probes (NG, DPA, EC, DBP, 2NDPA, AK2, 26DNT and PHE) in solution at a concentration of 10 mg L^−1^, with the exception of NG that was at a concentration of 100 mg L^−1^. Injection conditions were varied according to a full factorial design. This means that all possible combinations of the two parameters were experimentally tested, for a total of eight (= 2 × 4) different conditions. For each condition, five replicates were carried out. Outputs (i.e. peak areas) were plotted against the respective injection conditions and also assessed by type III analysis of variance (ANOVA). Type III (partial) sum of squares were preferred to type I and II mainly because it is a more robust choice for over-parameterised models and, therefore, a better alternative to correctly model the significant interaction effects that were observed between the two variables. To understand the full impact of the two parameters on the outputs, both the significance and size of their effects (main contributions and interaction) were assessed following best practices (see, for example, Sullivan & Feinn [25]). Specifically, the *p*-values observed after ANOVA *F*-test were used as a measure of effect significance, and the partial *η*^*2*^ (eta-squared) were determined as a measure of the effect size. Response curves were estimated through second-degree polynomial regression models. At the end, two sets of injection conditions (SICs) were selected for further performance evaluation. These involved the following parameters—SIC_1_: *L*_type_ = packed and *T*_inj_ = 260 °C and SIC_2_: *L*_type_ = empty and *T*_inj_ = 170 °C.

### Extraction of SLPs

SLP samples were directly recovered from live ammunition, kindly provided by the Northumbria Police Operational Tactical Training Centre (Newcastle upon Tyne, UK). Cartridges belonged to ten different ammunition types contained in as many ammunition boxes; the details are provided in Table 1.

**Table 1:** Main characteristics of the ammunition selected for this study

Ref.	Calibre	Brand	Bullet mass [gr]^a^	Bullet type^b^
A	.303 British	HXP	174	JHP
B	.303 British	Sako	180	JSP
C	9mm Parabellum	Sellier & Bellot	124	FMJ
D	9mm Parabellum	Speer	124	JHP
E	7.62mm Soviet	Sellier & Bellot	123	FMJ
F	7.62mm Soviet	Barnaul	123	FMJ
G	5.56mm NATO	NN	62	TMJ
H	5.56mm NATO	Magtech	62	FMJ
I	.40 S&W	Sellier & Bellot	180	FMJ
J	.30-30 Winchester	Federal	125	JHP

^a^Bullet masses are given in grains (1 gr = 64.80 mg)

^b^*FMJ* full metal jacket, *TMJ* total metal jacket, *JHP* jacket hollow point, *JSP* jacket soft point

From each ammunition box, two cartridges were opened using a kinetic hammer and the respective SLPs collected for analysis, for a total of 20 SLP samples. For each, 20 mg were weighed and transferred into a 15-mL glass centrifuge tube (Thermo Scientific, Loughborough, UK), where 2 mL of DCM was added. The tube opening was then closed using aluminium foil, vortexed for 10 s and left to extract for 3 h at room temperature in a fume cupboard [5, 24]. After extraction, the sample was vortexed again for 10 s and centrifuged for 5 min at 2000 rpm. An aliquot of 180 μL of the supernatant was then transferred to a GC vial equipped with a 200-μL insert, spiked with 20 μL of IS solution and put on the GC rack for GC-MS analysis.

Each SLP sample was analysed in duplicate, using each of the two SICs. For quantification, further dilutions were prepared, where needed. According to previous literature, all extraction procedures adopted in this work allowed nearly 100 % recovery for the most common SLP compounds. This has further been certified in-house through a series of preliminary tests (data not shown). A 100 % recovery was assumed for all the back-calculations and estimating the analyte concentrations in weighed SLP samples as mg g^−1^.

### Preliminary data analysis

A target ion and two qualifier ions were preliminarily selected for each of the 12 target analytes (Table S1). All acquired chromatograms were screened for these ions, and the presence of a target analyte assessed using the EC guidelines as identification criteria [26]. Identifications were thus accepted (1) if the deviations on the retention times (*t*_*R*_) were < 0.5 % compared to the reference standards and (2) if the deviations on the ion ratios were < 10% for those qualifier ions with a relative intensity of > 50% compared to the target ions, < 15% for those qualifier ions with a relative intensity of 20–50%, < 20% for those qualifier ions with a relative intensity of 10–20% and < 50% for those qualifier ions with a relative intensity of < 10%. As the purpose of this work was neither inter-laboratory validation or non-targeted analysis, conversion of *t*_*R*_ into retention indices has not been attempted.

For all identified analytes, peak areas (PAs) were obtained from the extracted ion chromatogram of the respective target ion and then further normalised to that of the IS when needed. For each SLP sample, a “chemical profile” composed of 12 features (compounds) was built by combining the observed normalised PAs for each of the 12 target analytes (target ions only). In some instances, the interdecile range (IDR) was used as an alternative measure of statistical dispersion: this has been defined as the range between the 1st and 9th deciles (D_1_ and D_9_, respectively).

### Performance evaluation

Limits of detection (LODs), limits of quantification (LOQs), linearity, precision and within-run drift were assessed for the two SICs through injection of solutions of the 12 target analytes. The ICH guidelines were followed [27]. Linearity was studied on a series of solutions spiked at 14 different concentrations between 1 and 5000 mg L^−1^, which were each analysed in replicates (*n* = 5). LODs and LOQs were estimated from the linear calibration curves of the different analytes and were respectively defined as 3 and 10 times the estimated standard deviations of PAs divided by the slope. Precision was assessed within-run and between-run using the relative standard deviation (%RSD) of the observed PAs, after the injection of ten replicates once per day over four days (*n* = 4 × 10). Three concentrations were tested (0.1, 1 and 10 μg·mL^−1^), with the exception of NG that was further tested at a concentration of 100 μg·mL^−1^. All working solutions were prepared in volumetric flasks using the appropriate solvent for the method assessed. Finally, within-run drift was studied on the QCs acquired during the analysis of the SLP samples (~200 analyses), in order to assess this parameter in true operational conditions. For each target analyte, a linear curve was fitted through the observed peak areas, and its relative rate of change (i.e. the ratio between the slope and the grand mean) was calculated.

### Further statistical assessment

The discriminating power (DP) was calculated as described by Gallidabino *et al.* [28]. For this, the chemical profiles observed for each replicate sample were first averaged and then compared, in order to determine the number of indistinguishable pairs (*m*). DPs were calculated both (1) after having taken into account the sole information brought by the presence/absence of the 12 target analytes (qualitative comparison) and (2) after having taken into account also the additional information brought by the normalised PAs (quantitative comparison). For the quantitative comparison, two SLPs were considered discriminated if, for at least one of their target analytes, the difference between the respective PAs was greater than 25%.

Association performances were primarily studied by principal component analysis (PCA) and hierarchical cluster analysis (HCA) of the single chemical profiles extracted from all the SLP samples. For HCA, specifically, an agglomerative nesting approach was used, and a number of similarity metrics were preliminarily tested, as well as different linkage criteria. Canberra distance (*d*_*C*_) and Ward’s method, respectively, were eventually selected because of their overall best results (data not shown). For initial visual assessment, the PCA score plots and HCA dendrograms were plotted. Then, the degree of cluster separation was more objectively calculated through the use of *ad hoc* metrics, i.e. the *J*_*2*_ index in PCA and agglomerative coefficient (AC) in HCA. The *J*_*2*_ indices were calculated as described by Gallidabino *et al.* [29]; only the first two principal components were taken into account. The ACs, on the contrary, were calculated as described by Kaufman and Rousseeuw [30]. To characterise the effect of the sampling error on the performance metrics, a leave-one-out (LOO) resampling strategy was used for model training. All the metrics, therefore, were initially determined on all LOO subsets and then averaged, in order to provide summary statistics.

The results of the similarity analysis step in HCA were further analysed to estimate the degree of accuracy in inferring whether, or not, two different samples come from the same source. For this, the *d*_*C*_ values observed during the pairwise comparison of all the samples were split into within-source and different-source groups, which were then studied by receiver operating characteristic (ROC) analysis. The area under the ROC curve was used as a summary metric of the overall classification performance. Accuracy was also calculated on the optimal cut-off threshold. This was defined as the point on the ROC curve closest to (1,1). A leave-two-out (LTO) resampling strategy was used to characterise the effect of the sampling error on the performance metrics.

### Software

Acquired chromatograms and MS spectra were analysed using Thermo Xcalibur v. 2.2, which was also used for PA extraction. R statistical computing software v. 4.1.3 was used for most subsequent data treatment. In particular, the following packages were adopted: “car” for ANOVA, “effectsize” for partial *η*^*2*^ calculations, “stats” for PCA and similarity metrics calculation, “hclust” and “cluster” for HCA and “pROC” for ROC analysis.

## Results and discussion

### Preliminary GC method optimisation and tests

**Fig. 1:**
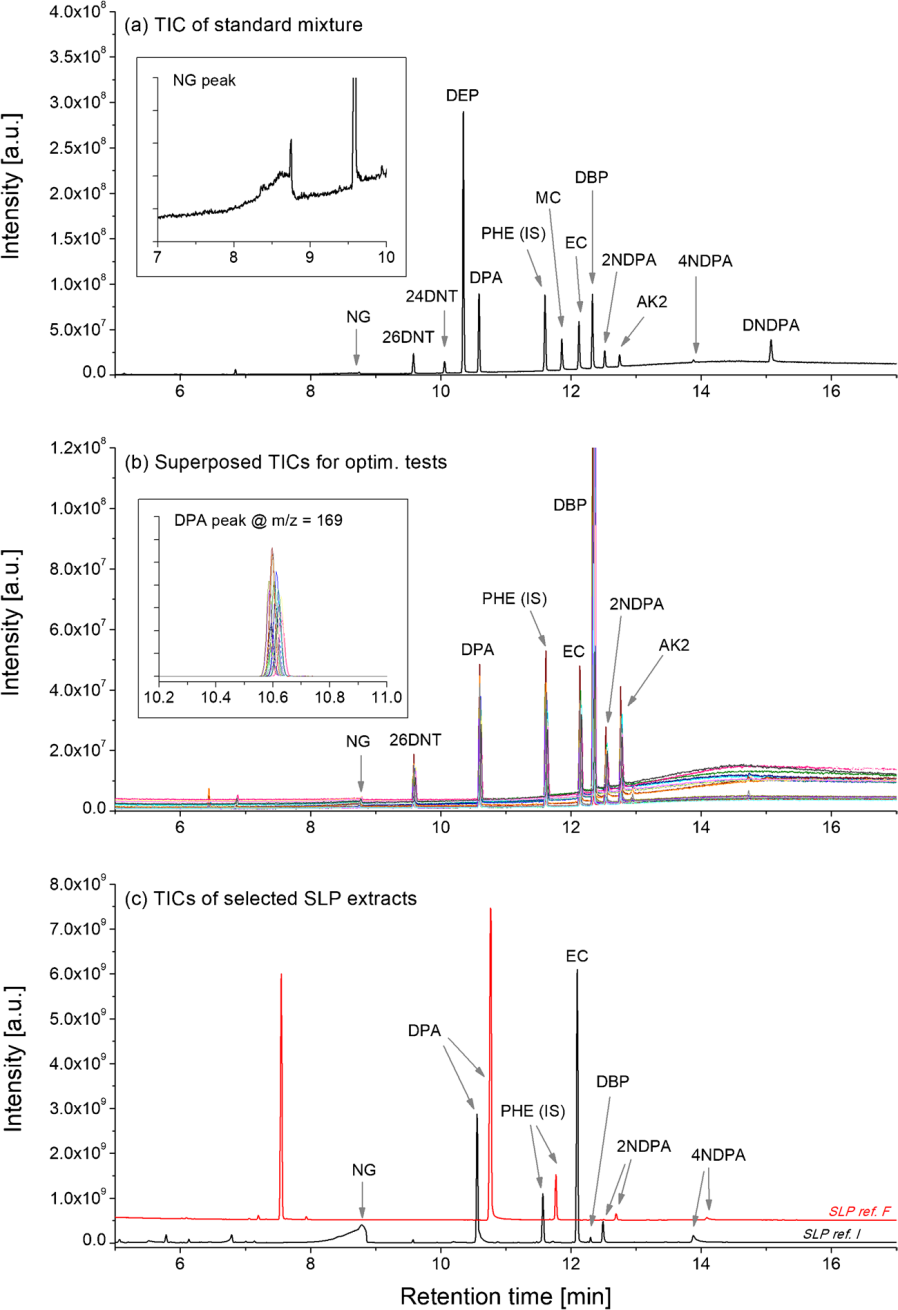
Total ion chromatograms (TICs) for (**a**) a mixed solution of the 12 target analytes plus the internal standard (IS) at a concentration of 10 mg L^−1^ analysed at *L*_type_ = packed and *T*_inj_ = 260 °C; (**b**) a mixed solution of 8 probes at a concentration of 10 mg L^−1^ analysed at all injection conditions tested in this work; (**c**) two SLP extracts, one from a single base SLP (ref. F) and one from a double base SLP (ref. I), analysed at *L*_type_ = packed and *T*_inj_ = 260 °C. In (**a**), the inset shows a closer look at the NG peak; in (**b**), the inset shows a closer look at the DPA peak after extraction of the *m/z* = 169 signal. In (**c**), the two TICs are offset by 0.2 min.

Twelve analytes typically found in SLP formulations were selected for this study, plus phenanthrene-d10 that was used as internal standard (IS), for a total of 13 compounds. Standard solutions were initially analysed at fixed injection conditions (*L*_type_ = packed and *T*_inj_ = 260 °C), in order to optimise the GC temperature program. The final method allowed complete separation of all 13 compounds within 20 min, without any co-elution (Fig. 1a). All peaks displayed good shapes, with little, or no, fronting/tailing. The only exception was NG, for which the peak presented a discernible fronting, probably due to injection-induced degradation. This observation aligned with previous works [5].

The injection conditions were varied and the effects on the resultant chromatograms assessed for a selection of eight probes. In particular, all combinations between two *L*_type_ (packed and empty) and four *T*_inj_ (170, 200, 230 and 260 °C) were tested, following a full factorial design (i.e. all the possible combinations were tested). Figure 1b shows an overlaid chromatogram of all the conditions tested. Important differences were observed for all probes at the level of their peak areas (PAs), which indicated a clear influence of one or both parameters on the inlet-to-column transfer yields. Variations in the retention times, half-height peak widths and peak shapes were negligible, providing only a minor impact on the overall separation efficiency. No other effects were observed at this stage. However, additional experiments on more concentrated standard solutions, and SLP extracts, revealed the appearance of peak tailing when *T*_inj_ ≤ 200 °C, which was typically limited to the later eluting analytes, especially (di)nitro-diphenylamines (Fig. S1). This was likely attributable to a slow analyte introduction to the column, which is characteristic of the use of a low inlet temperature [19]. The extend of this effect was marginal, even at the lower inlet temperatures, and, therefore, deemed negligible for practical purposes.

It is worth to note that injection tests were also carried out using MeOH as injection solvents. However, significant peak splitting was observed (Fig. S1). This was likely ascribable to the recondensation of MeOH into small droplets at the entry to the column, which can occur when MeOH is injected on a non-polar column (as the TG-5MS used in this work) and when the initial ramp temperature is set below its boiling point (i.e. 64.7 °C) [31]. Additional tests showed that the problem could be mitigated by increasing the initial oven temperature to 70 °C (i.e. over the boiling point of MeOH), but this was not explored further as this was beyond the scope of this work.

### Assessment of the parameter effects on the transfer yields

**Fig. 2:**
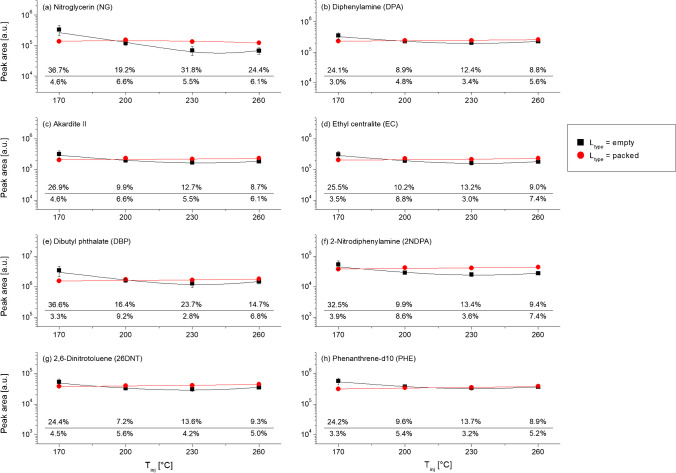
Response curves (peak areas vs. *T*_inj_) for the eight probes. The *y*-axis is on a log-10 scale. The respective measurement precisions, expressed in %RSDs (peak areas), are reported on the bottom of each image (above the line: *L*_type_ = empty; below the line: *L*_type_ = packed).

Figure 2 shows the response curves for all the probes. Overall, similar variations in the PAs as a result of changes in the injection conditions were observed across the eight probes, as seen by the similar shapes of the respective response curves. This suggested limited analyte-specific effects on the inlet-to-column transfer yields and, therefore, also the existence of optimal conditions within the tested experimental domain that were valid for all SLP compounds.

Results were compared. For each probe, PAs were seen to vary (even if at different extents and rates) as a function of both the inlet temperature and/or the liner type (Fig. 2), which proved that both parameters had a significant impact on the inlet-to-column transfer yields. Interestingly, however, the response curves were flatter when *L*_type_ = packed compared to when *L*_type_ = empty. This indicated that the influence of the inlet temperature, specifically, was strongly dependent on the liner type used, thereby also highlighting a significant interaction (= inter-dependency) between them. ANOVA was applied and further supported these conclusions (Table 2). Indeed, both the parameters were seen to have significant main effects and interactions on the signal intensity of all the probes (all *p* < 0.001). Interestingly, the largest effect sizes were typically observed for the *L*_type_ × *T*_inj_ interaction effects, thus proving that these were particularly influential on the inlet-to-column transfer yields (average *η*^*2*^ = 0.34 vs. *η*^*2*^ = 0.06 and 0.20 for *L*_type_ and *T*_inj_, respectively). Between the main effects, those associated with *T*_inj_ typically showed larger effect sizes than those associated with *L*_type_ which, therefore, also indicated that the inlet temperature was still more influential than the liner type. Perhaps as expected, however, the effect size for *T*_inj_ on the signal intensity of NG was particularly high compared to those observed on the signal intensities of the other compounds (*η*^*2*^ = 0.45 vs. *η*^*2*^ ≤ 0.23, respectively). This was surely because of high thermolability of NG and demonstrated its particular sensitivity on the liner temperature applied.

**Table 2:** Summary results for the statistical analysis of the effects (main contributions and interaction) of the liner type (*L*_type_) and inlet temperature (*T*_inj_) on the absolute peak areas of eight probes. *p*-Values observed after ANOVA *F*-test were used to examine the effect significance, i.e. whether or not any observed variation in the peak areas was due to chance: the lower the *p*-value, the more likely the effect was not due to chance. Partial *η*^*2*^ (eta squared) were used to examine the effect size, i.e. the relative magnitude of any observed variation in the peak areas (measured in terms of proportion of variance explained): the larger the partial *η*^*2*^, the greater the influence the parameter has

Compounds	*L*_*type*_	*T*_inj_	*L*_*type*_ × *T*_inj_
Nitroglycerin (NG)	*p* < 0.001 (***)^a^ *η*^*2*^ = 0.01 (^†^)^b^	*p* < 0.001 (***) *η*^*2*^ = 0.45 (^†††^)	*p* < 0.001 (***) *η*^*2*^ = 0.38 (^†††^)
Diphenylamine (DPA)	*p* < 0.001 (***) *η*^*2*^ = 0.01 (^†^)	*p* < 0.001 (***) *η*^*2*^ = 0.16 (^†††^)	*p* < 0.001 (***) *η*^*2*^ = 0.32 (^†††^)
Akardite II (AK2)	*p* < 0.001 (***) *η*^*2*^ < 0.01	*p* < 0.001 (***) *η*^*2*^ = 0.21 (^†††^)	*p* < 0.001 (***) *η*^*2*^ = 0.33 (^†††^)
Ethyl centralite (EC)	*p* < 0.001 (***) *η*^*2*^ = 0.02 (^†^)	*p* < 0.001 (***) *η*^*2*^ = 0.19 (^†††^)	*p* < 0.001 (***) *η*^*2*^ = 0.34 (^†††^)
Dibutyl phthalate (DBP)	*p* < 0.001 (***) *η*^*2*^ = 0.05 (^††^)	*p* < 0.001 (***) *η*^*2*^ = 0.23 (^†††^)	*p* < 0.001 (***) *η*^*2*^ = 0.32 (^†††^)
2-Nitrodiphenylamine (2NDPA)	*p* < 0.001 (***) *η*^*2*^ = 0.19 (^†††^)	*p* < 0.001 (***) *η*^*2*^ = 0.17 (^†††^)	*p* < 0.001 (***) *η*^*2*^ = 0.33 (^†††^)
2,6-Dinitrotoluene (26DNT)	*p* < 0.001 (***) *η*^*2*^ = 0.04 (^†^)	*p* < 0.001 (***) *η*^*2*^ = 0.09 (^††^)	*p* < 0.001 (***) *η*^*2*^ = 0.32 (^†††^)
Phenanthrene-d10 (PHE)	*p* < 0.001 (***) *η*^*2*^ = 0.19 (^†††^)	*p* < 0.001 (***) *η*^*2*^ = 0.13 (^††^)	*p* < 0.001 (***) *η*^*2*^ = 0.36 (^†††^)
MEANS	*p* < 0.001 *η*^*2*^ = 0.06	*p* < 0.001 *η*^*2*^ = 0.20	*p* < 0.001 *η*^*2*^ = 0.34
MEDIANS	*p* < 0.001 *η*^*2*^ = 0.03	*p* < 0.001 *η*^*2*^ = 0.18	*p* < 0.001 *η*^*2*^ = 0.33

^a^Significance codes: (***) < 0.001, (**) < 0.01, (*) < 0.05

^b^Size codes: (^†††^) ≥ 0.15 (large effect size), (^††^) ≥ 0.05 (medium effect size), (^†^) ≥ 0.01 (small effect size)

General trends were evaluated. When *L*_type_ = packed the effect of the liner temperature on the PAs was minimal, as seen by the mostly flat curves for all the probes (including NG). Still, the respective signal intensities were typically seen to slightly increase as a function of the temperature and reach a maximum at *T*_inj_ = 260 °C (Fig. 2). The exception was NG, for which the signal intensity was, instead, slightly better at lower temperatures (i.e. *T*_inj_ ≤ 200 °C). When *L*_type_ = empty, contrariwise, a maximum was generally seen at *T*_inj_ = 170 °C for all the probes. Perhaps as expected, NG presented the larger difference between this inlet temperature and any other temperature tested, which suggested that *T*_inj_ = 170 °C also allowed minimising heat-induced breakdowns. The highest PAs across conditions were generally observed for *L*_type_ = empty and *T*_inj_ = 170 °C. The latter were also the mildest in terms of potential heat transfer rate. Therefore, the findings aligned with previous literature that recommended minimising the heat transfer rate during injection, in order to minimise heat-induced breakdowns in the liner [5, 32]. But for this specific combination, however, response curves were predominantly superimposable and flat, thus indicating that the actual impact of the two parameters on the PAs was less significant than perhaps expected. Crucially, all probes were detected at good intensities and signal-to-noise ratios under all the conditions tested and no analyte showed a drastic drop in the respective PA at *T*_inj_ > 220 °C and/or when *L*_type_ = packed (Fig. 2). As a consequence, increasing the heat transfer rate through the use of a high temperature and/or a packed liner has less of a negative effect on the inlet-to-column transfer yields of the most thermolabile compounds when a traditional S/SL injector is employed than may be thought from previous studies [22, 24].

Regarding %RSDs, it was observed that the respective values at each inlet temperature were considerably larger when an empty liner was used compared to when a packed liner was used (average %RSD = 17.2 vs. 5.1 %, respectively). This finding, in combination with the mentioned discrepancy between the shapes of the response curves, suggested that the final inlet-to-column transfer yields for each probe were much more repeatable, as well as robust against variations in the injection conditions, when a packed liner was used. The better consistency of the PAs when *L*_type_ = packed was likely attributable to a quicker injection rate due to the larger heat capacity allowed by the quartz wool packing [33, 34]. Consequently, the liner type was shown, overall, to be a quite impactful variable, despite being largely unclarified in previously published works.

### Comparison of analytical method performances

Previous results showed that the use of injection conditions that allow low heat transfer rates was actually effective to minimise heat-induced degradations, but they were also prone to lead to less repeatable measurements. In this regard, the use of harsher injection conditions proved to enhance the repeatability of the measurements, without a drastic decrease in the analyte signal intensity. Therefore, these two approaches were further compared. Two sets of injection conditions (SICs) were selected, which corresponded to the harshest and mildest conditions in terms of heat transfer rate, respectively. These involved the following parameters—SIC_1_: *L*_type_ = packed and *T*_inj_ = 260 °C; and SIC_2_: *L*_type_ = empty and *T*_inj_ = 170 °C. The analytical performances of the two SICs were first assessed; Tables 3 and 4 report a summary of the characteristics measured, namely LOD/LOQ, precision, linearity and within-run drift.

**Table 3:** Main figures of merit for the two sets of injection conditions (SICs) compared in this work. Retention times (*t*_*R*_) were measured on 10 samples spiked at 10 mg L^−1^, but for NG and DNDPA, for which values were determined on 10 samples spiked at 100 mg L^−1^. For linearity, *R*^*2*^ values were determined up to a concentration of 10 mg L^−1^, but again for NG and DNDPA, for which values were determined up to a concentration of 100 mg L^−1^. The underlined values indicate the best results after direct comparison between SIC_1_ and SIC_2_

Compound	t_R_ [min]		LOD [mg L^-1^]^a^		LOQ [mg L^-1^]^a^		Linearity (R^2^) [-]		Within-run drift [%]	
	SIC_1_	SIC_2_	SIC_1_	SIC_2_	SIC_1_	SIC_2_	SIC_1_	SIC_2_	SIC_1_	SIC_2_
NG	8.77 ± 0.01	8.74 ± 0.01	2.03	4.80	6.78	16.00	0.9973	0.9876	−0.13	0.13
26DNT	9.60 ± 0.01	9.58 ± 0.01	0.17	0.53	0.55	1.75	0.9975	0.9964	−0.02	0.25
24DNT	10.08 ± 0.01	10.10 ± 0.01	0.16	2.14	0.54	7.12	0.9973	0.9939	−0.03	0.22
DEP	10.38 ± 0.01	10.34 ± 0.01	0.01	0.05	0.04	0.17	0.9973	0.9991	0.01	0.28
DPA	10.62 ± 0.01	10.64 ± 0.01	0.01	0.06	0.03	0.19	0.9928	0.9938	0.00	0.20
MC	11.89 ± 0.01	11.86 ± 0.01	0.13	0.44	0.45	1.47	0.9975	0.9963	0.00	0.24
EC	12.16 ± 0.01	12.12 ± 0.01	0.13	0.09	0.42	0.29	0.9973	0.9980	0.01	0.27
DBP	12.36 ± 0.01	12.33 ± 0.01	0.08	0.09	0.25	0.29	0.9939	0.9954	0.00	0.27
2NDPA	12.55 ± 0.01	12.55 ± 0.01	0.09	0.45	0.30	1.50	0.9931	0.9908	−0.01	0.33
AK2	12.78 ± 0.01	12.76 ± 0.01	0.10	0.45	0.34	1.50	0.9970	0.9995	−0.02	0.25
4NDPA	13.92 ± 0.01	13.97 ± 0.02	0.91	1.33	3.04	4.45	0.9945	0.9972	−0.10	0.33
DNDPA	15.16 ± 0.01	15.07 ± 0.01	2.71	5.45	9.04	18.16	0.9952	0.9938	−0.05	0.31
MEAN			*0.54*	*1.32*	*1.81*	*4.41*	*0.9959*	*0.9952*	−*0.03*	*0.26*
MEDIAN			*0.13*	*0.45*	*0.44*	*1.50*	*0.9972*	*0.9959*	−*0.02*	*0.26*

*LOD* limit of detection, *LOQ* limit of quantification

^a^Values can also be interpreted as (1) ng injected on column or (2) mg g^−1^ in weighted sample (assuming recovery = 100 %)

**Table 4:** Within-run and between-run precision (normalised PAs) for the two sets of injection conditions (SICs) compared in this work. An attempt was made to measure all values at three different concentrations (0.1, 1 and 10 mg L^−1^), but for NG, for which 100 mg L^−1^ was also tested. The underlined values indicate the best results after direct comparison between SIC_1_ and SIC_2_. Analytes are sorted by their retention time (*t*_*R*_)

Compound	Within-run precision (RSD) [%]								Between-run precision (RSD) [%]							
	SIC_1_				SIC_2_				SIC_1_				SIC_2_			
	@0.1^a^	@1	@10	@100	@0.1	@1	@10	@100	@0.1	@1	@10	@100a	@0.1	@1	@10	@100
NG	<LOD	<LOD	19.0	14.9	<LOD	<LOD	29.2	15.4	<LOD	<LOD	34.2	15.8	<LOD	<LOD	32.3	16.7
26DNT	<LOD	8.5	4.9	N/A^a^	<LOD	26.5	8.3	N/A	<LOD	10.6	5.1	N/A	<LOD	42.4	8.1	N/A
24DNT	<LOD	9.0	4.2	N/A	<LOD	<LOD	8.1	N/A	<LOD	11.8	4.6	N/A	<LOD	<LOD	8.6	N/A
DEP	5.7	6.8	6.7	N/A	32.5	10.3	7.4	N/A	6.3	7.1	6.5	N/A	38.4	9.8	7.2	N/A
DPA	5.8	5.4	4.6	N/A	30.4	41.7	5.2	N/A	7.5	5.6	4.7	N/A	33.3	40.6	7.2	N/A
MC	<LOD	6.5	2.8	N/A	<LOD	19.6	8.7	N/A	<LOD	7.3	3.1	N/A	<LOD	23.2	11.1	N/A
EC	<LOD	5.5	2.6	N/A	19.5	7.1	7.3	N/A	<LOD	6.3	3.0	N/A	31.7	10.2	10.7	N/A
DBP	13.1	8.8	5.9	N/A	36.3	7.9	11.3	N/A	23.8	10.3	6.1	N/A	50.4	8.9	11.2	N/A
2NDPA	7.9	7.7	6.0	N/A	<LOD	37.6	16.4	N/A	17.2	12.1	8.1	N/A	<LOD	40.9	17.9	N/A
AK2	<LOD	4.8	4.1	N/A	<LOD	19.4	11.7	N/A	<LOD	7.6	4.9	N/A	<LOD	24.4	14.7	N/A
4NDPA	<LOD	5.5	5.8	N/A	<LOD	<LOD	40.1	N/A	<LOD	13.9	6.8	N/A	<LOD	<LOD	47.8	N/A
DNDPA	<LOD	<LOD	9.4	N/A	<LOD	<LOD	18.9	N/A	<LOD	<LOD	35.7	N/A	<LOD	<LOD	18.9	N/A
MEAN	*8.1*	*6.8*	*6.3*	14.9	*29.7*	*21.2*	*14.4*	15.4	*13.7*	*9.3*	*10.2*	15.8	*38.5*	*25.0*	*16.3*	16.7
MEDIAN	*6.9*	*6.6*	*5.3*	14.9	*31.4*	*19.5*	*10.0*	15.4	*12.4*	*9.0*	*5.6*	15.8	*35.9*	*23.8*	*11.2*	16.7
MEAN (overall)	*7.1*				*19.0*				*10.6*				*22.7*			
MEDIAN (overall)	*5.9*				*16.4*				*7.3*				*17.9*			

*N/A* not determined, *<LOD* below limit of detection

^a^Values to be interpreted in mg L^−1^

Observed LODs typically ranged from 0.02 to 1.92 mg L^−1^ for SIC_1_ and from 0.06 to 4.53 mg L^−1^ for SIC_2_ (interdecile ranges). As a consequence, the two SICs were characterised by a similar analytical sensitivity, despite the higher signal intensities previously observed with SIC_2_ at the optimisation stage. This was likely a consequence of its lower measurement precision. More in general, the highest LODs were generally observed for the most thermolabile compounds and, specifically, NG and DNDPA (SIC_1_: 2.03 and 2.71 mg L^−1^; SIC_2_: 4.80 and 5.45 mg L^−1^). All the estimated values, however, were below the amounts commonly found in extracts from real samples, at least for the major SLP components, thus also indicating the suitability of both SICs for forensic applications. For each target analyte, differences were typically < 1 order of magnitude. Nonetheless, SIC_2_ yielded higher LODs for 11 of the 12 target analytes (i.e. all analytes, but EC), which highlighted the possibility of higher false-negative rates for determination of these compounds at trace levels. This could particularly affect the determination of (di)nitro-diphenylamines and dinitrotoluenes, which are commonly present at low concentration in SLPs. Interestingly, LODs for NG were slightly lower with SIC_1_ than SIC_2_ (i.e. 2.03 vs. 4.80 mg L^−1^, respectively), thus further emphasising that the mildest injection conditions did not automatically lead to the best results.

Precision was measured both in terms of within-run and between-run %RSDs. Within-run %RSDs typically ranged between 4.1 and 10.5% for SIC_1_ and between 7.3 and 37.0% with SIC_2_ (interdecile ranges)_._ Between-run %RSDs typically ranged between 4.7 and 19.2% with SIC_1_ and between 8.3 and 41.6% with SIC_2_ (interdecile ranges). As a consequence, SIC_1_ generally provided a better precision than SIC_2_, which was also observed at the optimisation stage. As mentioned, this was most likely due to a quicker injection rate, thanks to the higher inlet temperature used and, especially, the larger heat capacity allowed by the inlet quartz wool packing. More specifically, the lower %RSDs seen with SIC_1_ were systematically observed for all 12 target analytes, at any concentration level tested, with only few exceptions (i.e. between-precision for DBP at 1 mg L^−1^). Perhaps as expected, the highest %RSDs were observed at the lowest concentration levels, as well as for the most thermolabile compounds (i.e. NG, 2NDPA, 4NDPA and DNDPA), independently from the SIC applied. Still, SIC_1_ led to a milder increase in the %RSDs compared to SIC_2_, thereby also showing a more consistent precision between different SLP compounds and amounts injected. Overall, the precision level determined by SIC_1_ was deemed satisfactory for most applications, while that determined by SIC_2_ was sub-optimal and likely to lead to less accurate (semi-)quantitative measurements. Noteworthy, significantly better %RSDs were obtained on data normalised using the implemented IS, compared to non-normalised data, for both injection conditions (Table S3). This demonstrated a clear advantage to using an IS during the analyses to improve precision and was especially true for SIC_2_, which showed largely unsatisfactory precision levels before IS normalisation.

Linearity was investigated on log-transformed data. Independently from the injection conditions used, *R*^2^ values > 0.99 were observed for all target analytes, thereby supporting the good linearity of both sets of conditions on the studied concentration ranges. Differences between the two SICs were ≤ 0.003 and, therefore, not significant. The only exception was NG, for which a slightly worse linearity was observed when SIC_2_ was used (i.e. 0.9973 vs. 0.9876 for SIC_1_ and SIC_2_, respectively). Within-run drift was assessed to test any potential change in the inlet-to-column transfer yields over long GC runs, which could be indicative of a fast liner deterioration due to dirt accumulation and surface activation. This is particularly a matter of concern for the analysis of SLP extracts, due to the high sensitivity of related compounds to active sites [35]. Within-run drift was estimated on the QC datapoints acquired during the analysis of the SLP samples (which was ~ 200 analyses long). The two SICs showed very similar results, which were not indicative of any significant drift in the inlet-to-column transfer yields over time. Indeed, typical values ranged between −0.09 and 0.01% with SIC_1_ and between 0.20 and 0.32% with SIC_2_ (interdecile range).

### Application to the analysis and characterisation of SLPs

**Fig. 3:**
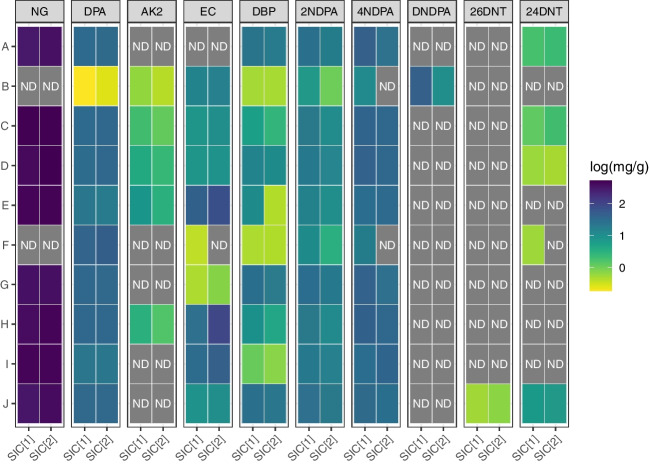
Heatmap comparing the compositions of the 10 SLPs analysed in this work, determined with each of the two sets of injection conditions (SICs). For each analyte, the intensity of the colour is proportional to the quantified concentrations in the samples (mg g^−1^), which is expressed on a log-10 scale. MC and DEP were not detected in any of the SLPs and, therefore, are not included in this image.

Samples from the 10 different SLPs were analysed with both SICs (see examples of chromatograms in Fig. 1c) and their composition initially determined. Amongst the 12 target analytes, 4 were observed in all the SLPs (i.e. DPA, 2NDPA, 4NDPA and DBP), 4 in ≥ 5 SLPs (i.e. NG, AK2, EC and 24DNT), 2 in just 1 SLP (i.e. DNDPA and 26DNT) and 2 in no SLP (i.e. MC and DEP). NG, in particular, was observed in 8 SLPs, indicating that they were double base, while the remaining were single base (Fig. 3). Where present, NG was always detected at concentrations > 320 mg g^−1^ (32%) and, therefore, was always the most concentrated compound (Table S4). The other analytes were detected at significantly lower levels, typically between 0.5 and 50 mg g^−1^ (0.05 and 5.0%). These results are in line with previous literature on SLP formulations [1, 36]. The particularly high concentrations of DNDPA in SLP ref. B indicated that this was in a relatively advanced state of ageing [37].

Results were compared. For each SLP, all analytes detected by SIC_2_ were also detected by SIC_1_, but the inverse was not true in four cases, which were classified as false negatives. These were EC in SLP ref. F, 4NDPA in SLPs ref. B and F and 24DNT in SLP ref. F (Fig. 3). For the latter situation (i.e. 24DNT in SLP ref. F), the estimated analyte concentration in the considered SLP was below the LODs achievable with SIC_2_, but not with SIC_1_. Therefore, the disparity was simply due to a difference in the analytical sensitivity. For the other three situations, the estimated concentrations were above the LODs achievable by both SICs, and, indeed, the respective target ions were detected in the chromatograms. However, their retention times and/or ion ratios did not match the reference values within the adopted identification criteria across the four replicates. As a consequence, the disparity was due to a difference in the identification accuracy, likely due to the worse precision of SIC_2_. From a quantitative point of view, the observed PAs for the all the target analytes, as well as their estimated concentrations, were largely comparable between the two SICs and correlated very well (Figs. S3 to S5). The within-cartridge %RSDs were also very similar, with values typically < 25% for normalised PA and < 15% for estimated concentrations (Fig S3 to S5). In particular, the observed within-cartridge %RSDs for the different analytes were largely comparable to those previously observed during the analytical performance assessment, at the concentrations actually measured in the SLPs. All these findings supported the reciprocal validity of the two SICs for the analysis and characterisation of SLPs, despite also highlighting the propensity of SIC_2_ to larger false detection rates towards minor and/or trace-level compounds.

Discrimination performances were evaluated. Independently from the SIC applied, the 10 analysed SLPs could be classified into 7 groups based on the sole information brought by the presence/absence of the 12 target analytes in their averaged chemical profiles. Three pairs (over the 45 possible) were initially not discriminated due to strong qualitative similarities (i.e. SLPs ref. C–D, E–H and G–I). These, however, could easily be resolved after having taken into account the additional information brought by the normalised PAs, bringing the final classification of the 10 analysed SLPs into 10 actual groups. Both SICs presented identical DPs, namely 93.3% (= 3 undisguisable pairs) at qualitative level and 100% (= 0 undisguisable pairs) at quantitative level. On one hand, these findings supported the hypothesis that the SLPs selected for this study were characterised by rather inter-variable compositions. On the other hand, they also showed that both SICs could successfully be used to distinguish them. Notably, the larger false detection rate previously observed with SIC_2_ did not particularly affect its DP, which also indicated that the significant discriminating information could be carried by a small selection of essential compounds. It is worth noting, however, that different SLP classes were inferred from the same samples depending on the SIC used, due to inconsistencies in the detection of the minor compounds. This could surely affect any cross-comparison of the results, which is an important issue to consider in an operational perspective and/or for the implementation of an inter-laboratory method.

### Comparison of forensic association performances

**Fig. 4:**
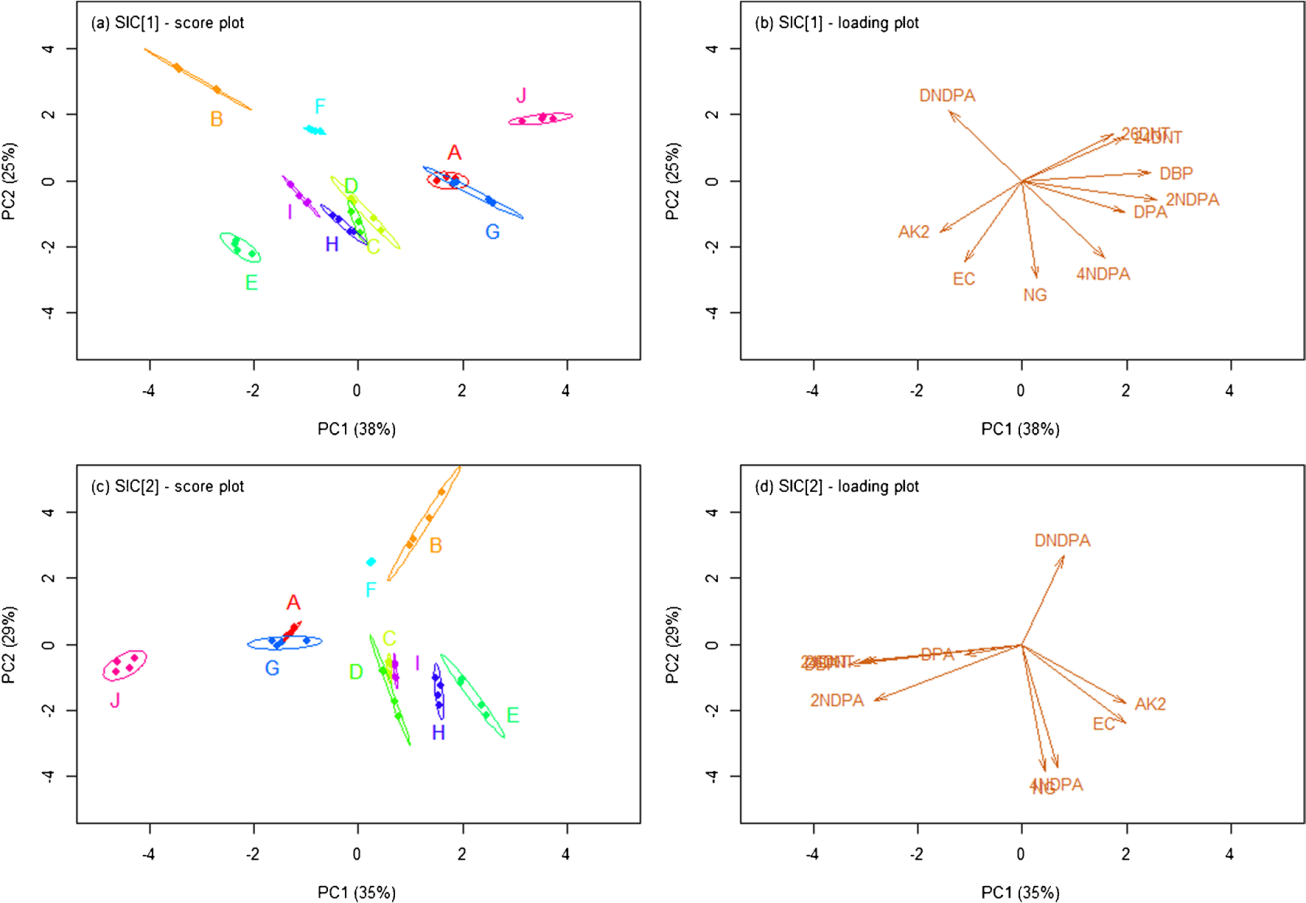
Score and loading plots after principal component analysis (PCA) of all SLP chemical profiles acquired with both sets of injection conditions (SICs) assessed in this work. The values in brackets in the axis labels are the percentages of explained variance on the first and second principal component (PC), respectively.

**Fig. 5:**
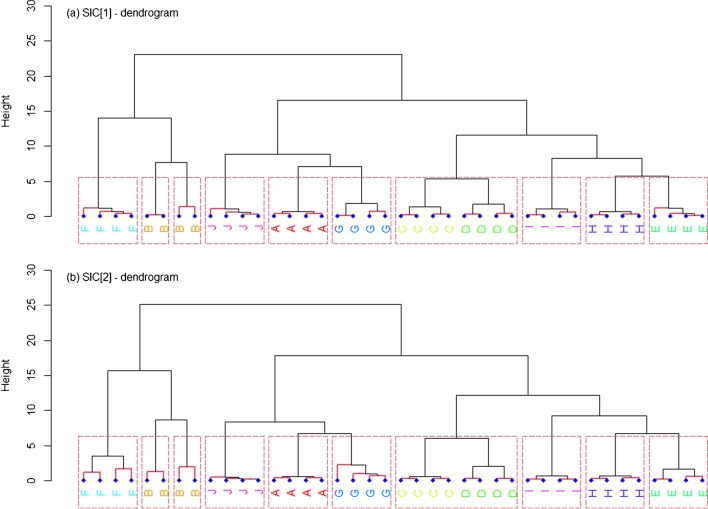
Dendrograms obtained after hierarchical cluster analysis (HCA) of all SLP chemical profiles acquired with both sets of injection conditions (SICs) assessed in this work. An agglomerative nesting approach was applied; Canberra distance and the Wards method were used as similarity metric and linkage criterion, respectively.

The original SLP classes are typically unknown in forensic casework. Hence, the ability of both SICs to infer whether, or not, two samples came from a common source was studied using unsupervised classification methods, namely PCA and HCA. The related outputs (i.e. PCA score plots and HCA dendrograms) are shown in Figs. 4 and 5. Again, very similar results were observed between the two SICs, with no meaningful differences in the grouping performances. In particular, the single chemical profiles for the 10 analysed SLPs tended to form well resolved clusters in the respective PCA score plots and HCA dendrograms that, in most of the cases, were not superposed (or only partially superposed). The *J*_*2*_ index and AC were used as metrics to quantively characterise the cluster quality for PCA and HCA, respectively, through the use of a resampling strategy. The average *J*_*2*_ index in PCA (first 2 PCs only) was 7.86E3 ± 1.51E3 and 2.74E3 ± 4.60E2 for SIC_1_ and SIC_2_, respectively, while the average AC in HCA was 0.979 ± 0.002 and 0.973 ± 0.002. All these values were relatively large, supporting a good clustering of the single chemical profiles in both PCA and HCA, independently from the SIC used.

On closer inspection, the pairs formed by SLPs ref. E–H and G–I led to well discriminated clusters on both the PCA score plots and HCA dendrograms, even if they were previously shown to share the same qualitative compositions (Figs. 4 and 5). This was likely due to significant quantitative differences in the respective analyte concentrations, which was further supported by the analysis of the PCA loadings. On the contrary, no complete discrimination was observed for the clusters of SLPs ref. C–D, surely because of their similar compositions at both qualitative and quantitative levels. A similar situation was observed for SLPs ref. A–G, at least in PCA (Fig. 4). The latter observation, however, was not expected, due to the SLPs showing significant differences already at qualitative level. On further analysis, the partial superposition of the respective clusters was likely ascribable to the small amounts of 24DNT and EC in their chemical profiles that led to a negligible impact on the between-source variability. The same pair, nonetheless, was well discriminated in HCA which, therefore, proved to be less affected by minor changes at quantitative level. Interestingly, the clustering quality was shown to be strongly SLP-dependent. Indeed, the cluster sizes in PCA and branch heights in HCA varied between different SLPs, thus also indicating a highly variable within-source variability (Figs. 4 and 5). In this regard, SLP ref. B was characterised by the largest within-source variability between the measurements, while SLP ref. F by the lowest. As highlighted above, SLP ref. B was the only powder presenting evidence of an advanced state of ageing, and, consequently, its particularly high within-source variability was attributable to slightly inhomogeneous ageing conditions between the different samples analysed. On the HCA dendrograms, this translated into a split between samples when grouping was forced (Fig. 5).

**Fig. 6:**
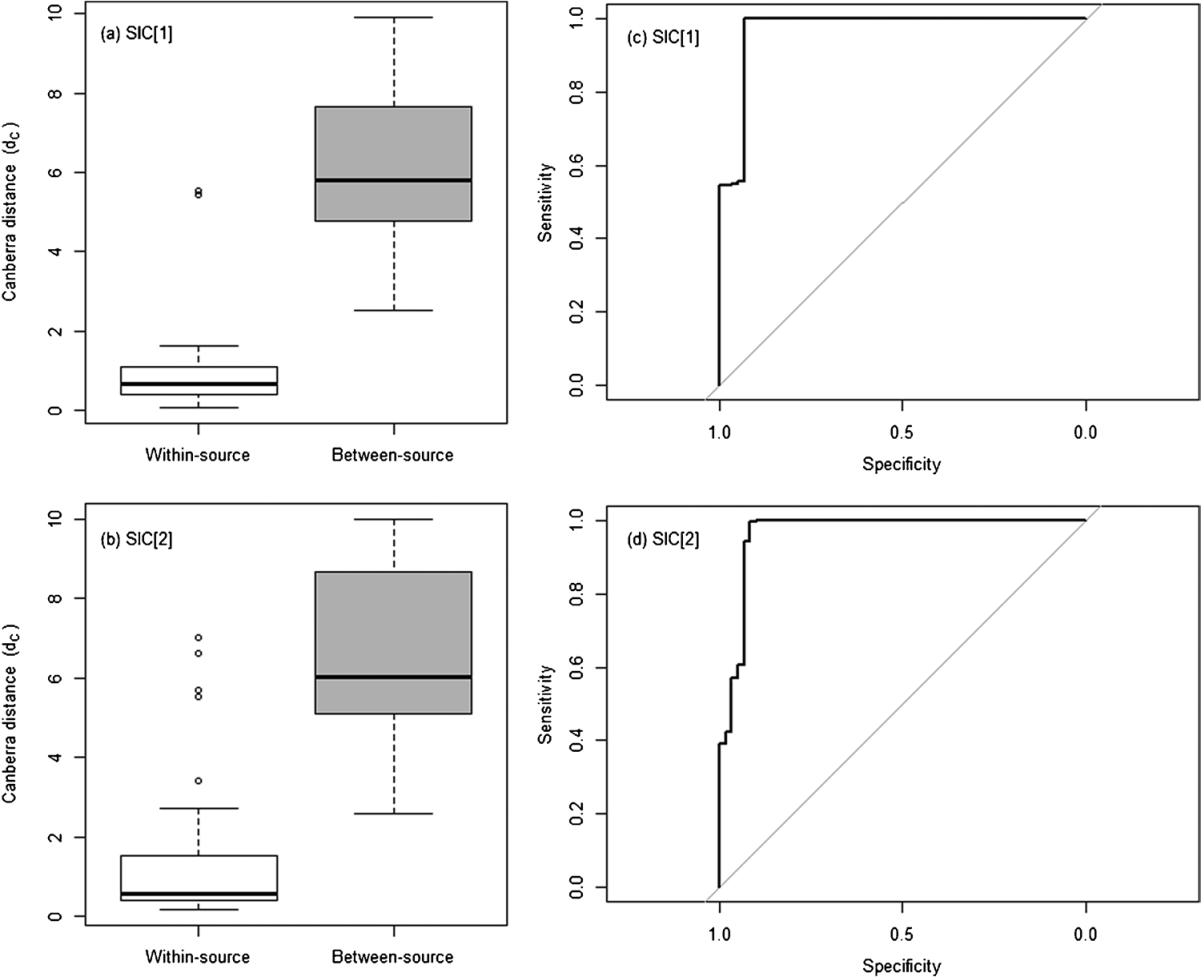
Summary of the results obtained after pairwise comparison using Canberra distance (*d*_*C*_) of all SLP chemical profiles acquired with both sets of injection conditions (SICs) assessed in this work. In particular: (**a**) and (**b**) show the boxplots of the within-source and between-source distributions of *d*_*C*_ values, and (**c**) and (**d**) show the respective receiver operating characteristic (ROC) curves. The grey lines on the two ROC curves indicate the performance of a random classifier, as a reference.

As similarity analysis is a very common strategy to identify potential links between samples in forensic applications (e.g. [38]), the results of the pairwise comparison step carried out prior to HCA were analysed more in depth. Canberra distance (*d*_*C*_) was used as a similarity metric. Within-source *d*_*C*_ values typically ranged between 0.266 and 1.433 with SIC_1_, and between 0.284 and 2.544 with SIC_2_ (interdecile ranges), while between-source *d*_*C*_ values typically ranged between 3.590 and 9.178 with SIC_1_, and between 3.718 and 9.577 with SIC_2_ (interdecile ranges). As expected, within-source comparisons generally led to significantly lower *d*_*C*_ values compared to between-source comparisons, thus denoting a higher level of agreement between the chemical profiles from the same SLP (Fig. 6a-b). For each SIC, the difference between the means of the respective distributions was statistically significant according to the Welch’s *t*-test (all *p* < 0.001). Still, no perfect discrimination between them was observed in both cases, highlighting that any selected decision cut-off would lead to a classification accuracy < 100 %. Noteworthy, the within-source comparison of the chemical profiles for SLP ref. B led to particularly high *d*_*C*_ values that were in the variation range of those observed in between-source comparisons.

ROC analysis was applied to study the classification performances for varying cut-offs (Fig. 6c-d). The average AUC was 0.970 ± 0.006 and 0.966 ± 0.007 for SIC_1_ and SIC_2_, respectively, while the average accuracy was 99.5 ± 0.1% and 97.4 ± 2.2% at the optimal cut-off. As a consequence, the two SICs only showed negligible differences in association performance, despite the mentioned inconsistencies in the detection of the minor compounds.

## Conclusion

In this work, the parameters potentially affecting the sample injection in GC-MS analysis of SLPs using a traditional S/SL injector were assessed for their effects on the analytical and sample association performances. These were the liner type (*L*_type_) and inlet temperature (*T*_inj_). Results showed that both could potentially affect the exhaustiveness and repeatability of the observed chemical profiles, with *L*_type_ being particularly sensitive despite typically not being clarified in published works. Perhaps as expected, degradation effects were observed for the most thermolabile compounds (e.g. nitroglycerin) at conditions maximising the heat transfer rates (*L*_type_ = packed and *T*_inj_ ≥ 200 °C). However, these did not seem to be as drastic as, perhaps, suggested in previous studies. Indeed, (1) all target analytes were still detected at acceptable signal-to-noise ratios when the harshest injection conditions were used (i.e. *L*_type_ = packed and at *T*_inj_ = 260 °C) and (2) similar analytical and comparison performances were observed between these conditions and the mildest ones (i.e. *L*_type_ = empty and at *T*_inj_ = 170 °C). Yet, the harshest conditions allowed for enhanced within-run and between-run precision compared to the mildest ones, as well as offering a slightly better overall analytical sensitivity, that led to a lower false negative rate for the detection of minor compounds in SLPs.

As a conclusion, data collected in this study suggested that minimising the heat transfer rate through the use of a low inlet temperature and, eventually, an empty liner is not necessarily synonymous with better performances in the forensic analysis of SLPs using GC-MS and a traditional S/SL injector. While these conditions are surely valid for the characterisation of the major compounds in SLPs for general purposes, future works aiming at advanced profiling applications (e.g. class attribution and source association) should preferentially consider the use of a packed liner and an inlet temperature of 260 °C, in order to take advantage of the extra information brought by the detection of minor compounds and an enhanced measurement precision. Given its importance on the method analytical performances and subsequent comparison of the extracted chemical profiles, it is also suggested that future works explicitly report the liner type used, in order to have a better understanding of the potential of the method proposed.

### Supplementary Information

Below is the link to the electronic supplementary material.

Supplementary file1 (DOCX 677 KB)
