# Correction to: Forensic profiling of smokeless powders (SLPs) by gas chromatography–mass spectrometry (GC‑MS): a systematic investigation into injector conditions and their effect on the characterisation of samples

**DOI:** 10.1007/s00216-024-05218-8

**Published:** 2024-03-07

**Authors:** Blake Kesic, Niamh McCann, Samantha L. Bowerbank, Troy Standley, Jana Liechti, John R. Dean, Matteo D. Gallidabino

**Affiliations:** 1 Department of Applied Sciences, Northumbria University, Newcastle Upon Tyne, NE1 8ST UK; 2 King’s Forensics, Department of Analytical, Environmental & Forensic Sciences, King’s College London, London, SE1 9NH UK

**Correction to: Analytical and Bioanalytical Chemistry**

10.1007/s00216-024-05189-w

The following ORCID iDs were missing in the published article:

0000-0001-7972-0868 of Blake Kesic, 0000-0002-4685-9350 of Samantha L. Bowerbank, 0009-0006-7219-3720 of Troy Standley, 0000-0001-6118-9079 of Jana Liechti, and 0000-0001-5729-356x of John R. Dean.

