# Supplementary material for: Forensic profiling of smokeless powders (SLPs) by gas chromatography–mass spectrometry (GC-MS): a systematic investigation into injector conditions and their effect on the characterisation of samples
Source: Anal Bioanal Chem. 2024 Feb 9;416(8):1907–22. doi: 10.1007/s00216-024-05189-w (PMC10901999; doi:10.1007/s00216-024-05189-w)
Supplement: Supplementary file 1 — Supplementary file1 (DOCX 677 KB) [file 216_2024_5189_MOESM1_ESM.docx]

Electronic Supplementary Material to

**Forensic profiling of smokeless powders (SLPs) by gas chromatography – mass spectrometry (GC-MS): a systematic investigation into injector conditions and their effect on the characterisation of samples**

Blake Kesic, Niamh McCann, Samantha L. Bowerbank, Troy Standley, Jana Liechti, John R. Dean, Matteo D. Gallidabino*

*Email: [matteo.gallidabino@kcl.ac.uk](mailto:matteo.gallidabino@kcl.ac.uk)

Table of contents

S-2: List of target analytes

S-3: Effects of peak deteriorating conditions on TICs

S-4: Precision of non-normalised peak areas

S-5: Estimated analyte concentrations in the 10 SLPs

S-6: Comparison of the observed peak areas and related RSDs

S-7: Comparison of the estimated concentrations and related RSDs

S-8: Correlation between analytes in SLPs

List of target analytes

**Table S1** – Analytes targeted in this work, with related CAS registry numbers and retention times (*t_R_*) observed with the GC method used. The internal standard (IS) is reported at the end of the list.

| ***#*** | ***Analyte*** | ***Abbrev.*** | ***t_R_ [min]*** | ***Target ion*** | ***Qualifiers ions*** | ***CAS*** | ***Supplier*** | ***Purity*** |
| --- | --- | --- | --- | --- | --- | --- | --- | --- |
| 1 | *Nitroglycerin* | NG | 8.77 | 46 | 58, 76 | 55-63-0 | Thames Restek | 99 % |
| 2 | *2,6-Dinitrotolune* | 26DNT | 9.60 | 165 | 63, 89 | 606-20-2 | Sigma Aldrich | 97 % |
| 3 | *2,4-Dintrotoluene* | 24DNT | 10.08 | 165 | 63, 89 | 121-14-2 | Sigma Aldrich | 97 % |
| 4 | *Diethyl phthalate* | DEP | 10.38 | 149 | 150, 177 | 84-66-2 | Sigma Aldrich | 99.5 % |
| 5 | *Diphenylamine* | DPA | 10.62 | 169 | 167, 168 | 122-39-4 | Fisher Scientific | 99 % |
| 6 | *Methyl centralite* | MC | 11.89 | 134 | 106, 240 | 611-92-7 | Fisher Scientific | 99 % |
| 7 | *Ethyl centralite* | EC | 12.16 | 120 | 148, 268 | 85-98-3 | Sigma Aldrich | 99 % |
| 8 | *Dibutyl phthalate* | DBP | 12.36 | 149 | 150, 223 | 84-74-2 | Sigma Aldrich | 99 % |
| 9 | *2-Nitrodiphenylamine* | 2NDPA | 12.55 | 214 | 167, 180 | 119-75-5 | Sigma Aldrich | 98 % |
| 10 | *Akardite II* | AK2 | 12.78 | 169 | 167, 168 | 13114-72-2 | Sigma Aldrich | > 95 % |
| 11 | *4-Nitrodiphenylamine* | 4NDPA | 13.92 | 214 | 167, 168 | 836-30-6 | Fisher Scientific | 98 % |
| 12 | *2,4-Dinitrodiphenylamine* | DNDPA | 15.16 | 259 | 166, 167 | 961-68-2 | Sigma Aldrich | 98 % |
| IS | *Phenanthrene-d10* | PHE | 11.61 | 188 | 184, 189 | 1517-22-2 | Sigma Aldrich | 98 % |

Effects of peak deteriorating conditions on TICs


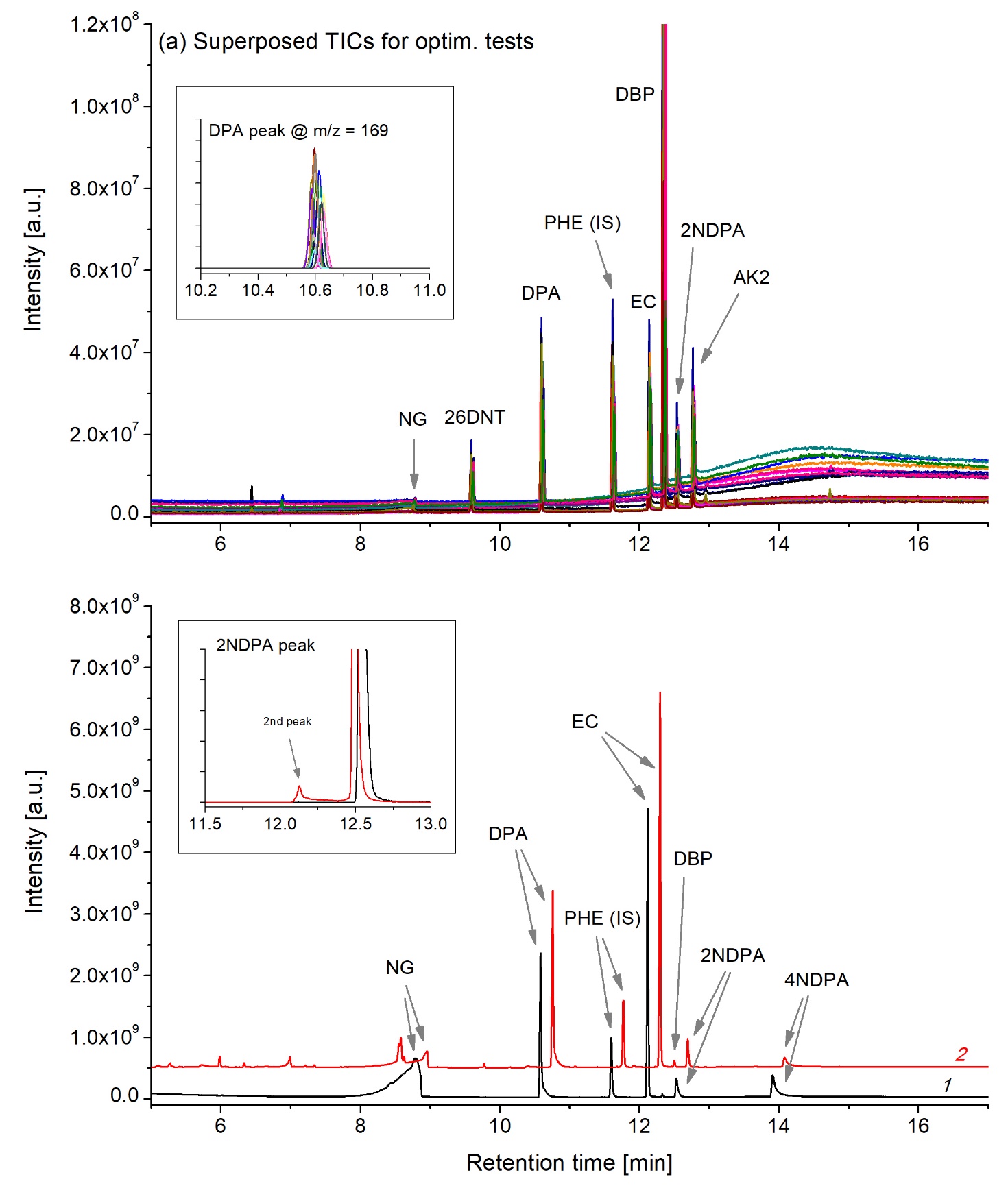


**Figure S1** – Total ion chromatograms (TICs) showing the same SLP (ref. I) analysed at different injection conditions, i.e. (1) *L*_type_ = empty and *T*_inj_ = 170 ºC using DCM as injection solvent and (2) *L*_type_ = packed and *T*_inj_ = 260 ºC using MeOH as injection solvent. The inset shows a closer look at the 2NDPA peak. Peak tailing was visible on the late eluting analytes when conditions a low injection temperature were used, while peak splitting was visible on all the analytes when MeOH was used as injection solvent. The two TICs are offset by 0.2 min.

Precision of non-normalised peak areas

**Table S3** – Within-run and between-run precision (absolute PAs) for the two sets of injection conditions (SICs) compared in this work. There was an attempt to measure all nvalues at three different concentrations, i.e. 0.1, 1 and 10 mg L^-1^, but for NG, for which 100 mg L^-1^ was also tested. Analytes are sorted by their retention time (*t_R_*).

| ***Compound*** | ***Within-run precision (RSD)***  ***[%]*** | | | | | | | | ***Between-run precision (RSD)***  ***[%]*** | | | | | | | |
| --- | --- | --- | --- | --- | --- | --- | --- | --- | --- | --- | --- | --- | --- | --- | --- | --- |
|  | ***SIC_1_*** | | | | ***SIC_2_*** | | | | ***SIC_1_*** | | | | ***SIC_2_*** | | | |
|  | ***@0.1 mg L^-1^*** | ***@1 mg L^-1^*** | ***@10 mg L^-1^*** | ***@100 mg L^-1^*** | ***@0.1 mg L^-1^*** | ***@1 mg L^-1^*** | ***@10 mg L^-1^*** | ***@100 mg L^-1^*** | ***@0.1 mg L^-1^*** | ***@1 mg L^-1^*** | ***@10 mg L^-1^*** | ***@100 mg L^-1^*** | ***@0.1 mg L^-1^*** | ***@1 mg L^-1^*** | ***@10 mg L^-1^*** | ***@100 mg L^-1^*** |
| *NG* | <LOD | <LOD | 21.0 | 16.8 | <LOD | <LOD | 26.3 | 23.0 | <LOD | <LOD | 33.5 | 18.1 | <LOD | <LOD | 33.0 | 24.6 |
| *26DNT* | <LOD | 7.3 | 5.2 | N/A | <LOD | 70.2 | 13.1 | N/A | <LOD | 9.3 | 5.8 | N/A | <LOD | 72.5 | 21.5 | N/A |
| *24DNT* | <LOD | 7.5 | 4.8 | N/A | <LOD | 41.0 | 21.4 | N/A | <LOD | 10.1 | 5.4 | N/A | <LOD | 41.5 | 27.7 | N/A |
| *DEP* | 6.1 | 3.5 | 2.3 | N/A | 39.1 | 13.9 | 16.0 | N/A | 6.1 | 3.9 | 2.6 | N/A | 45.5 | 23.4 | 23.5 | N/A |
| *DPA* | 10.2 | 5.0 | 9.5 | N/A | 32.7 | 49.6 | 37.2 | N/A | 10.3 | 4.9 | 8.7 | N/A | 34.4 | 51.1 | 46.7 | N/A |
| *MC* | <LOD | 4.5 | 11.5 | N/A | <LOD | 18.5 | 36.3 | N/A | <LOD | 4.7 | 10.9 | N/A | <LOD | 40.2 | 46.8 | N/A |
| *EC* | 10.0 | 4.3 | 11.1 | N/A | 33.0 | 26.3 | 37.0 | N/A | 10.6 | 4.8 | 10.7 | N/A | 42.4 | 34.2 | 48.0 | N/A |
| *DBP* | 12.3 | 5.7 | 2.7 | N/A | 32.9 | 15.3 | 18.2 | N/A | 22.5 | 6.3 | 3.3 | N/A | 55.9 | 26.7 | 23.7 | N/A |
| *2NDPA* | 13.5 | 6.1 | 15.1 | N/A | <LOD | 41.1 | 44.9 | N/A | 21.8 | 9.9 | 15.1 | N/A | <LOD | 55.8 | 55.7 | N/A |
| *AK2* | <LOD | 5.7 | 13.5 | N/A | <LOD | 25.3 | 41.7 | N/A | <LOD | 7.9 | 13.2 | N/A | <LOD | 44.8 | 52.3 | N/A |
| *4NDPA* | <LOD | 6.5 | 14.0 | N/A | <LOD | 48.3 | 70.2 | N/A | <LOD | 14.3 | 13.4 | N/A | <LOD | 56.5 | 87.3 | N/A |
| *DNDPA* | <LOD | <LOD | 12.3 | N/A | <LOD | <LOD | 26.9 | N/A | <LOD | <LOD | 38.8 | N/A | <LOD | <LOD | 35.1 | N/A |
|  | | | | | | | | | | | | | | | | |
| *MEAN* | 10.5 | 5.6 | 10.2 | 16.8 | 34.4 | 32.5 | 32.4 | 23.0 | 15.2 | 7.6 | 13.4 | 18.1 | 44.5 | 43.6 | 41.8 | 24.6 |
| *MEDIAN* | 11.2 | 5.7 | 11.3 | 16.8 | 33.0 | 25.8 | 31.6 | 23.0 | 16.1 | 7.1 | 10.8 | 18.1 | 43.9 | 42.5 | 40.9 | 24.6 |
| *MEAN (overall)* | *8.8* | | | | *32.4* | | | | *11.7* | | | | *42.1* | | | |
| *MED. (overall)* | *7.3* | | | | *32.7* | | | | *9.9* | | | | *42.4* | | | |

“N/A”: not determined; “<LOD”: below respective limit of detection.

Estimated analyte concentrations in the 10 SLPs

**Table S4** – Estimated analyte concentrations in the 10 SLPs analysed in this study, determined with SIC_1_. Values are expressed in mg g^-1^ and were back-calculated assuming a recovery of 100%. MC and DEP were not detected in any of the SLPs and are, therefore, not included in this table.

| ***Powder (ref.)*** | ***NG*** | ***DPA*** | ***AK2*** | ***EC*** | ***DBP*** | ***2NDPA*** | ***4NDPA*** | ***DNDPA*** | ***26DNT*** | ***24DNT*** |
| --- | --- | --- | --- | --- | --- | --- | --- | --- | --- | --- |
| *A* | 322.8  ± 14.4 | 34.8  ± 0.4 | ND^a^ | ND | 25.3  ± 0.6 | 27.9  ± 0.6 | 46.5  ± 1.6 | ND | ND | 1.8  ± 0.3 |
| *B* | ND | 0.2  ± 0 | 0.7  ± 0.0 | 14.1  ± 14.5 | 0.5  ± 0.1 | 7.3  ± 6.0 | 11.9  ± 8.7 | 46.7  ± 14.1 | ND | ND |
| *C* | 508.5  ± 62.1 | 35.3  ± 1.2 | 2.0  ± 0.1 | 8.0  ± 0.3 | 5.6  ± 0.9 | 20.9  ± 1.1 | 42.6  ± 2.4 | ND | ND | 1.3  ± 0.3 |
| *D* | 461.8  ± 50.7 | 32.7  ± 1.0 | 4.1  ± 0.3 | 7.8  ± 0.9 | 14.2  ± 0.8 | 21.5  ± 0.4 | 44.1  ± 1.0 | ND | ND | 0.6  ± 0.0 |
| *E* | 481.5  ± 52.8 | 22.9  ± 0.5 | 8.1  ± 0.4 | 45.7  ± 6.3 | 11.3  ± 0.6 | 15.4  ± 0.2 | 31.9  ± 0.6 | ND | ND | ND |
| *F* | ND | 41.5  ± 1.9 | ND | <LOQ | 0.5  ± 0.0 | 11.4  ± 0.1 | 18.7  ± 0.7 | ND | ND | 0.6  ± 0.0 |
| *G* | 369.5  ± 39.2 | 39.1  ± 1.0 | ND | 0.5  ± 0.1 | 29.7  ± 0.6 | 31.9  ± 2.5 | 43.8  ± 2.5 | ND | ND | ND |
| *H* | 403.1  ± 17.6 | 37.3  ± 0.9 | 3.3  ± 0.1 | 29.4  ± 0.4 | 9.5  ± 0.6 | 20.8  ± 0.9 | 45.6  ± 3.5 | ND | ND | ND |
| *I* | 494.3  ± 31.4 | 23.9  ± 0.5 | ND | 32.9  ± 0.7 | 1.2  ± 0.1 | 21.3  ± 1.0 | 31.9  ± 3.2 | ND | ND | ND |
| *J* | 343.1  ± 43.3 | 35.8  ± 0.5 | ND | 8.8  ± 0.1 | 28.0  ± 5.2 | 29.0  ± 0.6 | 33.6  ± 1.0 | ND | 0.6  ± 0.0 | 6.9  ± 0.3 |
|  | | | | | | | | | | |
| *MEAN* | 423.1 | 30.3 | 3.6 | 16.4 | 12.6 | 20.7 | 35.1 | 46.7 | 0.6 | 2.2 |
| *MEDIAN* | 432.4 | 35.0 | 3.3 | 8.8 | 10.4 | 21.1 | 38.1 | 46.7 | 0.6 | 1.3 |

"ND”: not detected (i.e., <LOD); “<LOQ”: below respective limit of quantitation.

Comparison of the observed peak areas and related RSDs

**Figure S3** – Comparison of the (a) average peak areas (n = 4) and (b) related relative standard deviations (RSDs) for each target analyte in the 10 SLPs, determined using each of the two sets of injection conditions (SICs) tested in this work. MC and DEP were not detected in any of the SLPs and are, therefore, not included. DNDPA and 26DN were detected in only one SLP, hence the lack of errors bars.

Comparison of the estimated concentrations and related RSDs

**Figure S4** – Comparison of the (a) average estimated concentrations (n = 4) and (b) related relative standard deviations (RSDs) for each target analyte in the 10 SLPs, determined using each of the two sets of injection conditions (SICs) tested in this work. MC and DEP were not detected in any of the SLPs and are, therefore, not included. DNDPA and 26DN were detected in only one SLP, hence the lack of errors bars.

Correlation between analytes in SLPs


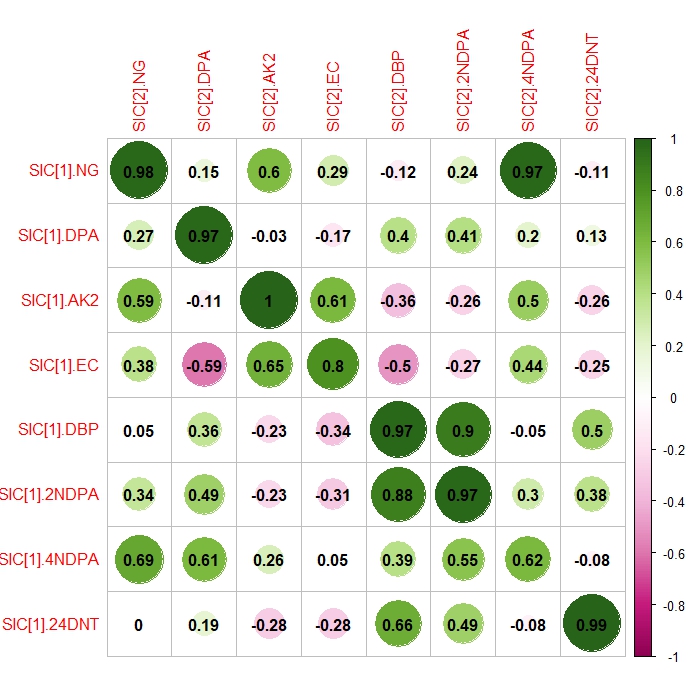


**Figure S5** – Plot of the Pearson`s correlation coefficients (PCCs) determined between the peaks areas of all the pairs of compounds detected in SLPs using each of the two sets of injection conditions (SICs) tested in this work. MC and DEP were not detected in any of the SLPs, while DNDPA and 26DNT were only detected in one SLP. Therefore, these were excluded from the analysis.
